# Development and validation of the PET-CT score for diagnosis of malignant pleural effusion

**DOI:** 10.1007/s00259-019-04287-7

**Published:** 2019-03-22

**Authors:** Min-Fu Yang, Zhao-Hui Tong, Zhen Wang, Ying-Yi Zhang, Li-Li Xu, Xiao-Juan Wang, Wan Li, Xiu-Zhi Wu, Wen Wang, Yu-Hui Zhang, Tao Jiang, Huan-Zhong Shi

**Affiliations:** 1 Department of Nuclear Medicine, Beijing Institute of Respiratory Medicine and Beijing Chao-Yang Hospital, Capital Medical University, Beijing, China; 2 Department of Respiratory and Critical Care Medicine, Beijing Institute of Respiratory Medicine and Beijing Chao-Yang Hospital, Capital Medical University, 8 Gongti Nanlu, Chao-Yang District, Beijing, 100020 China; 3 Department of Radiology, Beijing Institute of Respiratory Medicine and Beijing Chao-Yang Hospital, Capital Medical University, Beijing, China

**Keywords:** Computed tomography, Malignant pleural effusion, Positron emission tomography

## Abstract

**Purpose:**

Although some parameters of positron emission tomography with ^18^F-fluorodeoxyglucose (^18^F-FDG) and computed tomography (PET-CT) are somehow helpful in differentiating malignant pleural effusion (MPE) from benign effusions, no individual parameter offers sufficient evidence for its implementation in the clinical practice. The aim of this study was to establish the diagnostic accuracy of a scoring system based on PET-CT (the PET-CT score) in diagnosing MPE.

**Methods:**

One prospective derivation cohort of patients with pleural effusions (84 malignant and 115 benign) was used to develop the PET-CT score for the differential diagnosis of malignant pleural effusion. The PET-CT score was then validated in another independent prospective cohort (*n* = 74).

**Results:**

The PET-CT parameters developed for discriminating MPE included unilateral lung nodules and/or masses with increased ^18^F-FDG uptake (3 points); extrapulmonary malignancies (3 points); pleural thickening with increased ^18^F-FDG uptake (2 points); multiple nodules or masses (uni- or bilateral lungs) with increased ^18^F-FDG uptake (1 point); and increased pleural effusion ^18^F-FDG uptake (1 point). With a cut-off value of 4 points in the derivation cohort, the area under the curve, sensitivity, specificity, positive likelihood ratio, and negative likelihood ratio of the PET-CT score to diagnose MPE were 0.949 (95% CI: 0.908–0.975), 83.3% (73.6%–90.6%), 92.2% (85.7%–96.4%), 10.7 (5.6–20.1), and 0.2 (0.1–0.3), respectively.

**Conclusions:**

A simple-to-use PET-CT score that uses PET-CT parameters was developed and validated. The PET-CT score can help physicians to differentiate MPE from benign pleural effusions.

**Electronic supplementary material:**

The online version of this article (10.1007/s00259-019-04287-7) contains supplementary material, which is available to authorized users.

## Introduction

Malignant pleural effusion (MPE) is frequently observed in multiple malignancies, with lung cancer being the most frequent underlying malignancy [1, 2]. As the prognosis for patients with MPE is poor [3, 4], an efficacious procedure that can establish a definite diagnosis as early as possible with a minimum risk and discomfort is highly desirable. Series examining the diagnostic rate for malignancy of pleural cytology has reported a mean sensitivity of about 60% (range 40–87%), which has highlighted the challenge of MPE diagnosis [5, 6]. It is important to avoid subjecting frail patients to unnecessarily invasive procedures and to select just those who may benefit the most from such interventions.

Positron emission tomography (PET) with ^18^F-fluorodeoxyglucose (^18^F-FDG) was reported for the first time to be an effective tool in the evaluation of pleural diseases in 1997 by Bury et al. [7]. The subsequent four prospective studies showed that ^18^F-FDG PET and computed tomography (CT) can be a useful method for the differential diagnosis of MPE [8–11]. However, these four prospective studies recruited small numbers of patients with pleural effusions, and the evidence supporting the discriminatory role of ^18^F-FDG PET-CT was not validated in an independent population. One recent meta-analysis suggested that no individual ^18^F-FDG PET imaging measurement appears to predict the probability of MPE enough to be recommended in the routine work-up for effusions of undetermined cause [12]. We undertook the present prospective study to develop a scoring system based on ^18^F-FDG PET-CT findings (the PET-CT score) for clinicians to discriminate MPE from benign effusion.

## Methods

### Study populations

From May 1, 2014 through October 31, 2015, all consecutive adult patients admitted to the Department of Respiratory and Critical Care Medicine of Beijing Chaoyang Hospital, China who underwent investigation of exudative pleural effusions and ^18^F-FDG PET-CT were enrolled in the derivation cohort. From November 1, 2015 to October 31, 2016, all consecutive adult patients with pleural effusions who underwent ^18^F-FDG PET-CT at the Department of Nuclear Medicine of Beijing Chaoyang Hospital were enrolled in a validation cohort. For both derivation and validation cohorts, obtaining a definite cause of the pleural effusion was required for final inclusion into the study.

Following STARD guidelines, this study was conducted in accordance with the Declaration of Helsinki and approved by the institutional ethics committee of Beijing Chaoyang Hospital, Beijing, China (ID 2014-ke-98). Patients provided written informed consent.

### Diagnostic criteria

The diagnosis of MPE was established if malignant cells were detected upon cytological examination of the pleural fluid or biopsy specimens that were obtained during the same admission with the PET-CT examination. Tuberculous pleural effusion was diagnosed when Ziehl–Neelsen stains or Lowenstein–Jensen cultures of pleural fluid, sputum, or pleural biopsy specimens were positive or when granulomas were found in the parietal pleural biopsies. A parapneumonic effusion was defined as any effusion associated with bacterial pneumonia, lung abscess, bronchiectasis, or empyema, when reported with the presence of pus within the pleural space. Other causes of pleural effusions followed well-established clinical criteria. The patients were followed up for at least 12 months to ensure the absence of malignant pleural processes if they were diagnosed to have benign effusion [13].

### PET-CT imaging

The integrated ^18^F-FDG PET-CT study was performed on a GE Discovery STE device using a standard protocol before invasive procedures were performed. All patients fasted for at least 6 h and had a blood glucose level of <200 mg/dL before ^18^F-FDG administration. Whole body PET-CT scans were acquired 55–73 min (mean 63.2 ± 7.3 min) after intravenous injection of 3.7 MBq/kg of ^18^F-FDG. A body scan from the skull base to the upper thighs was firstly performed and then was followed by a head scan. CT parameters for body scan were: 140 kV, 120 mA, and slice thickness of 3.75 mm. CT parameters for head scan were: 120 kV, 200 mA, and slice thickness of 3.75 mm. PET parameters were: 2.5 min/bed for body scan and 5 min/bed for head scan in 3-dimension mode. Attenuation-corrected PET images (voxel size: 3.9 × 3.9 × 3.3-mm for both body and head scan) were reconstructed using a 3-dimensional ordered-subset expectation maximization algorithm (14 subsets and 2 iterations for body scan, and 28 subsets and 2 iterations for head scan). Integrated PET and CT images were obtained automatically on AW VolumeShare2 (GE Healthcare).

One radiologist (TJ) and one nuclear physician (MFY) evaluated the PET-CT images together and a final consensus was obtained on all imaging findings. Both observers were blinded to the final diagnosis of pleural effusion. Pleural ^18^F-FDG uptake was calculated by manually drawing regions of interest (ROIs) on PET and CT registered images slice by slice, and the maximum standardized uptake value (SUVmax) was selected to represent ^18^F-FDG uptake. ^18^F-FDG uptake of pleural fluid was evaluated on the registered slice with the deepest fluid. A circular ROI of 5 mm diameter was placed 5 mm to the parietal pleura, and the SUVmax of pleural fluid was recorded. To obtain a background value of ^18^F-FDG uptake, mediastinal uptake was measured by placing an ROI on the superior vena cava and the SUVmean was recorded. All SUVs were normalized to body weight. Then, a target-to-background ratio (TBR) was determined by calculating the ratio of the SUVmax of the pleura or pleural fluid, and the SUVmean of the mediastinum. Lung nodules and/or masses (diameter ≥ 8 mm) were identified, and the SUVmax was measured. Lymph nodes with high uptake (higher than that in the surrounding normal soft tissues) but without calcification and without attenuation higher than 70 HU were regarded as positive. Other organs were classified as positive when there was focal ^18^F-FDG uptake, compared with the surrounding normal organ (tissue) or ^18^F-FDG uptake that could not be explained by physiologic activity.

Continuous data of PET-CT features (pleural ^18^F-FDG uptake, pleural effusion ^18^F-FDG uptake, and pleural effusion depth) were first transferred to categorical data by means of drawing the receiver-operating-characteristic (ROC) curves and calculating the cut-off values. Pleural effusion depth was measured in centimeters for maximum anteroposterior depth of the effusion on chest CT scans. The cut-off value of the SUVmax of lung nodule was set as 2.5.

PET-CT parameters evaluated for the discriminating analysis included: (1) pleural thickening (≥3 mm); (2) pleural nodule(s) (≥1 cm); (3) increased pleural ^18^F-FDG uptake (TBR > 1.8); (4) pleural thickening (≥3 mm) with increased ^18^F-FDG uptake (TBR > 1.8); (5) pleural nodule(s) with increased uptake (TBR > 1.8); (6) pleural calcifications; (7) unilateral effusion; (8) massive pleural effusion (depth > 16.5 cm); (9) increased pleural effusion ^18^F-FDG uptake (TBR > 1.1); (10) pleural loculations (i.e., an effusion that is compartmentalized, has septations or a convex shape facing the lung parenchyma, or accumulated in a nondependent portion); (11) lung nodules and/or masses; (12) lung nodules and/or masses with increased ^18^F-FDG uptake (SUVmax ≥ 2.5); (13) lung single nodule or mass with increased ^18^F-FDG uptake (SUVmax ≥ 2.5); (14) multiple nodules or masses (uni- or bilateral lungs) with increased ^18^F-FDG uptake (SUVmax ≥ 2.5); (15) unilateral lung nodules and/or masses with increased ^18^F-FDG uptake (SUVmax ≥ 2.5); (16) bilateral lung nodules and/or masses with increased 18F-FDG uptake (SUVmax ≥ 2.5); (17) obstructive atelectasis or pneumonia (i.e., FDG uptake was visibly increased at the obstruction site of the bronchus, regardless of the FDG uptake within the distal collapsed lung); (18) mediastinal positive lymph node(s); (19) unilateral positive hilar lymph node(s); (20) bilateral positive hilar lymph node(s); (21) positive hilar lymph node(s); (22) extra-thoracic positive lymph node(s); (23) pericardial effusion; (24) pericardial effusion with increased ^18^F-FDG uptake (TBR > 1.1); (25) cardiomegaly (cardiothoracic ratio > 0.5 in axial images); (26) dilation of the inferior vena cava (diameter > 1.7 cm, measured just above the entrance of the suprahepatic veins); (27) ascites; (28) ascites with increased ^18^F-FDG uptake (TBR > 1.1); and (29) extrapulmonary malignancies (primary/metastatic).

### Statistical analysis

Categorical and continuous data were expressed as numbers (percentages) and means ± SD, respectively. Between-group comparisons were performed with *X*^2^, Fisher’s exact, and Student’s *t* tests, as appropriate. We calculated differences between groups by using ANOVA and univariate analysis, entering only variables with a *p* value < 0.01 into the multiple regression models. A logistic regression analysis with backward conditional method served to select those imaging variables entering the scoring system. Weight values to each variable were assigned proportionally to the magnitude of the logistic equation’s coefficients.

ROC curves were drawn and the areas under the curve (AUCs) were calculated to determine the diagnostic value of the PET-CT measurement or score, including sensitivity, specificity, positive likelihood ratio, and negative likelihood ratio [14, 15]; and AUCs were compared using z-statistic with the Hanley and McNeil procedure [16]. The optimum cut-off values were defined based on their maximum Youden index (sensitivity + specificity − 1). The parameters of diagnostic accuracy are reported together with their 95% confidence intervals (CIs). To verify the diagnostic accuracy of the PET-CT score in the validation cohort, cases with the PET-CT score above the cut-off value obtained in the derivation cohort were considered as positive results. Interobserver agreement about the PET-CT parameters that make up the PET-CT score was evaluated using κ statistics. The statistical significance level was set at 0.05 (two-tailed). All analyses were conducted with MedCalc and SPSS version 23.0 statistical software.

## Results

### Study populations

**Fig. 1:**
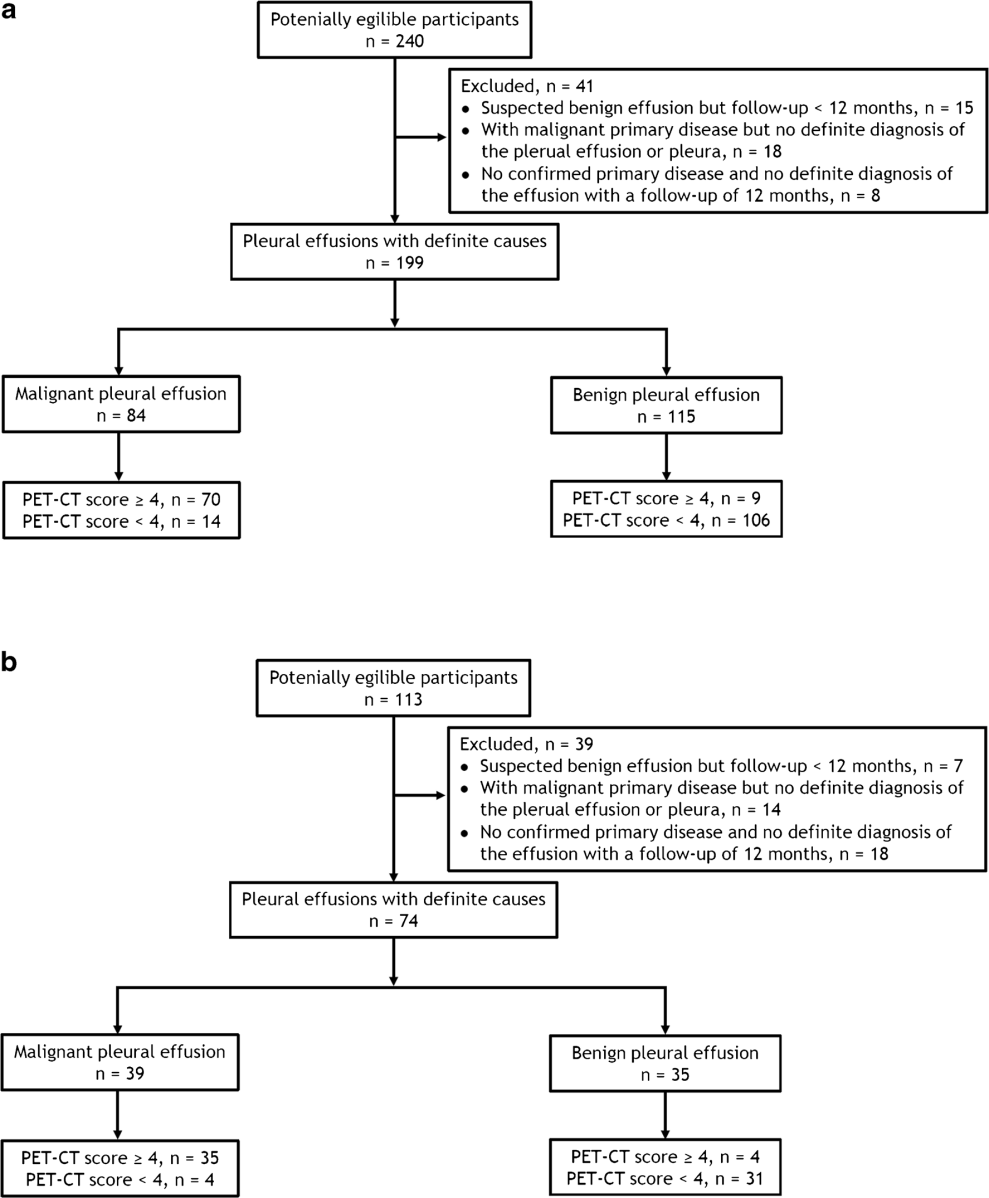
Flow diagrams of study populations in the derivation cohort (**a**) and in the validation cohort (**b**)

As shown in Fig. 1, 41 patients in the derivation cohort and 39 in the validation cohort were excluded for the following reasons: (1) suspected benign effusion but follow-up <12 months; (2) with malignant primary disease but no definite diagnosis of the plerual effusion or pleura during the same admission with the PET-CT examination; and (3) no confirmed primary disease and no definite diagnosis of the effusion with a follow-up of 12 months. The characteristics and diagnostic work-up of the excluded patients were presented in Supplemental Table 1. Eventually, a total of 199 patients were included in the derivation cohort and 74 patients in the validation cohort, and their baseline characteristics are shown in Table 1. There were 113 males and 86 females in the derivation cohort, and the average age of the patients was 60.3 ± 16.4 years (range, 21–88 years). In the validation cohort, 43 patients were males and 31 females, and the average age was 61.7 ± 15.3 years (range, 18–90 years).

**Table 1:** Baseline characteristics of patients with pleural effusion (*n*, %)

Characteristics	Derivation cohort (N = 199)	Validation cohort (N = 74)	*p* value
Age, year	60.3 ± 16.4	61.7 ± 15.3	0.509
Sex			0.844
Male	113 (56.8)	43 (58.1)	
Female	86 (43.2)	31 (41.9)	
Malignant pleural effusion	84 (42.2)	39 (52.7)	0.137
Lung cancer	65 (77.4)	25 (64.1)	
Malignant mesothelioma	6 (7.1)	2 (5.1)	
Breast cancer	3 (3.5)	0	
Lymphoma	2 (2.4)	4 (10.3)	
Ovarian cancer	2 (2.4)	3 (7.7)	
Pancreatic cancer	1 (1.2)	0	
Unknown origin^a^	5 (6.0)	5 (12.8)	
Benign pleural effusion	115 (57.8)	35 (47.3)	<0.001
Tuberculosis	57 (49.6)	7 (20.0)	
Parapneumonic effusion	46 (40.0)	15 (42.9)	
Empyema	3 (2.6)	0	
Pulmonary embolism	3 (2.6)	6 (17.1)	
Pneumosilicosis	2 (1.7)	3 (8.6)	
Chylothorax	1 (0.9)	1 (2.9)	
Parasitic infection	1 (0.9)	0	
Nonspecific pleurisy	2 (1.7)	3 (8.6)	

^a^Malignant cells were present in pleural fluid but the original sites of primary tumors could not be identified

Patients in the derivation cohort were similar to those in the validation cohort in terms of the distribution of age, sex, and etiology of MPE. Differences in the etiological distribution of benign pleural effusions were found between the two cohorts (X^2^ = 23.56, *p* < 0.001, Table 1). A total of 42.2% of the patients in the derivation cohort and 52.7% of the patients in the validation cohort were confirmed to have MPE; 57.8% of the patients in the derivation cohort and 47.3% of the patients in the validation cohort suffered from benign pleural effusion (Table 1). The most frequent cause of MPE is lung cancer, followed by mesothelioma in both cohorts. The percentage of tuberculous pleural effusion in the derivation cohort was significantly higher than that in the validation cohort (49.6% vs. 20.0%, *p* = 0.002).

### Development of the PET-CT score from the derivation cohort

All PET and CT images were qualified. Initially, we evaluated 29 PET-CT parameters in the bivariate analysis using univariate analysis, and 19 of them displayed statistical significance with *p* < 0.01 in the discriminating analysis (Table 2). These 19 PET-CT variables were further processed using a multivariable logistic regression model. The model selected five variables that were predictive of malignancy, which were included to establish weight scores as follows: (1) unilateral lung nodules and/or masses with increased ^18^F-FDG uptake (SUVmax ≥ 2.5) (3 points); (2) extrapulmonary malignancies (primary/metastatic) (3 points); (3) pleural thickening (≥3 mm) with increased ^18^F-FDG uptake (TBR > 1.8) (2 points); (4) multiple nodules or masses (uni- or bilateral lungs) with increased ^18^F-FDG uptake (SUVmax ≥ 2.5) (1 point); and (5) increased pleural effusion ^18^F-FDG uptake (TBR > 1.1) (1 point). For the above variables comprising the PET-CT score, interobserver agreement of measurement was perfect or substantial, with κ coefficients of 0.965, 0.981, 0.888, 0.914, and 0.760, respectively. Thus, the PET-CT scores ranged from 0 to 10 (Table 3).

**Table 2:** Univariate analysis of PET/CT scan findings in the derivation cohort (*n*, %)

Characteristics	Malignant effusion (N = 84)	Benign effusion (N = 115)	OR (95% CI)	*p* values
Pleural thickening (≥3 mm)	83 (98.8)	84 (73.0)	30.6 (4.1–229.6)	<0.001
Pleural nodule(s) (≥1 cm)	46 (54.8)	15 (13.0)	8.1 (4.0–16.1)	<0.001
Increased pleural ^18^F-FDG uptake (TBR > 1.8)	71 (84.5)	42 (36.5)	9.5 (4.7–19.2)	<0.001
Pleural thickening (≥3 mm) with increased ^18^F-FDG uptake (TBR > 1.8)	71 (84.5)	41 (35.7)	9.9 (4.9–19.9)	<0.001
Pleural nodule(s) with increased uptake (TBR > 1.8)	46 (54.8)	14 (12.2)	8.7 (4.3–17.7)	<0.001
Unilateral effusion	73 (86.9)	69 (60.0)	4.4 (2.1–9.2)	<0.001
Massive pleural effusion (depth > 16.5 cm)	16 (19.0)	4 (3.5)	6.5 (2.1–20.3)	<0.001
Increased pleural effusion ^18^F-FDG uptake (TBR > 1.1)	43 (51.2)	35 (30.4)	2.4 (1.3–4.3)	0.003
Lung nodules and/or masses	65 (77.4)	17 (14.8)	19.7 (9.5–40.7)	<0.001
Lung nodules and/or masses with increased ^18^F-FDG uptake (SUVmax ≥ 2.5)	61 (72.6)	13 (11.3)	20.8 (9.8–44.1)	<0.001
Lung single nodule or mass with increased ^18^F-FDG uptake (SUVmax ≥ 2.5)	22 (26.2)	7 (6.1)	5.5 (2.2–13.5)	<0.001
Multiple nodules or masses (uni- or bilateral lungs) with increased ^18^F-FDG uptake (SUVmax ≥ 2.5)	39 (46.4)	6 (5.2)	15.7 (6.2–39.8)	<0.001
Unilateral lung nodules and/or masses with increased ^18^F-FDG uptake (SUVmax ≥ 2.5)	52 (61.9)	8 (7.0)	21.7 (9.4–50.5)	<0.001
Obstructive atelectasis or pneumonia	22 (26.2)	1 (0.9)	40.5 (5.3–307.3)	<0.001
Mediastinal positive lymph node(s)	46 (54.8)	41 (35.7)	2.2 (1.2–3.9)	0.009
Unilateral positive hilar lymph node(s)	31 (36.9)	5 (4.3)	12.9 (4.7–35.0)	<0.001
Positive hilar lymph node(s)	41 (48.8)	33 (28.7)	2.4 (1.3–4.3)	<0.001
Extra-thoracic positive lymph node(s)	30 (35.7)	16 (13.9)	3.4 (1.7–6.9)	<0.001
Extrapulmonary malignancies (primary/metastatic)	44 (52.4)	4 (3.5)	30.5 (10.3–90.4)	<0.001

*CI* confidence interval, ^*18*^*F-FDG*^18^F-fluorodeoxyglucose, *OR* odds ratio, *SUV* standardized uptake value, *TBR* target-to-background ratio

**Table 3:** Development of the PET-CT score for diagnosing malignant pleural effusion from the derivation cohort

Parameter	OR (95% CI)	Score
Unilateral lung nodules and/or masses with increased ^18^F-FDG uptake (SUVmax ≥ 2.5)	49.7 (10.6–233.2)	3
Extrapulmonary malignancies (primary/metastatic)	49.0 (9.8–244.3)	3
Pleural thickening (≥3 mm) with increased ^18^F-FDG uptake (TBR > 1.8)	9.8 (3.0–31.0)	2
Multiple nodules or masses (uni- or bilateral lungs) with increased ^18^F-FDG uptake (SUVmax ≥ 2.5)	3.0 (1.4–6.4)	1
Increased pleural effusion ^18^F-FDG uptake (TBR > 1.1)	3.4 (1.2–9.6)	1

*CI* confidence interval, ^*18*^*F-FDG*^18^F-fluorodeoxyglucose, *OR* odds ratio, *SUV* standardized uptake value, *TBR* target-to-background ratio

### Diagnostic performance of the PET-CT score in the derivation cohort

**Fig. 2:**
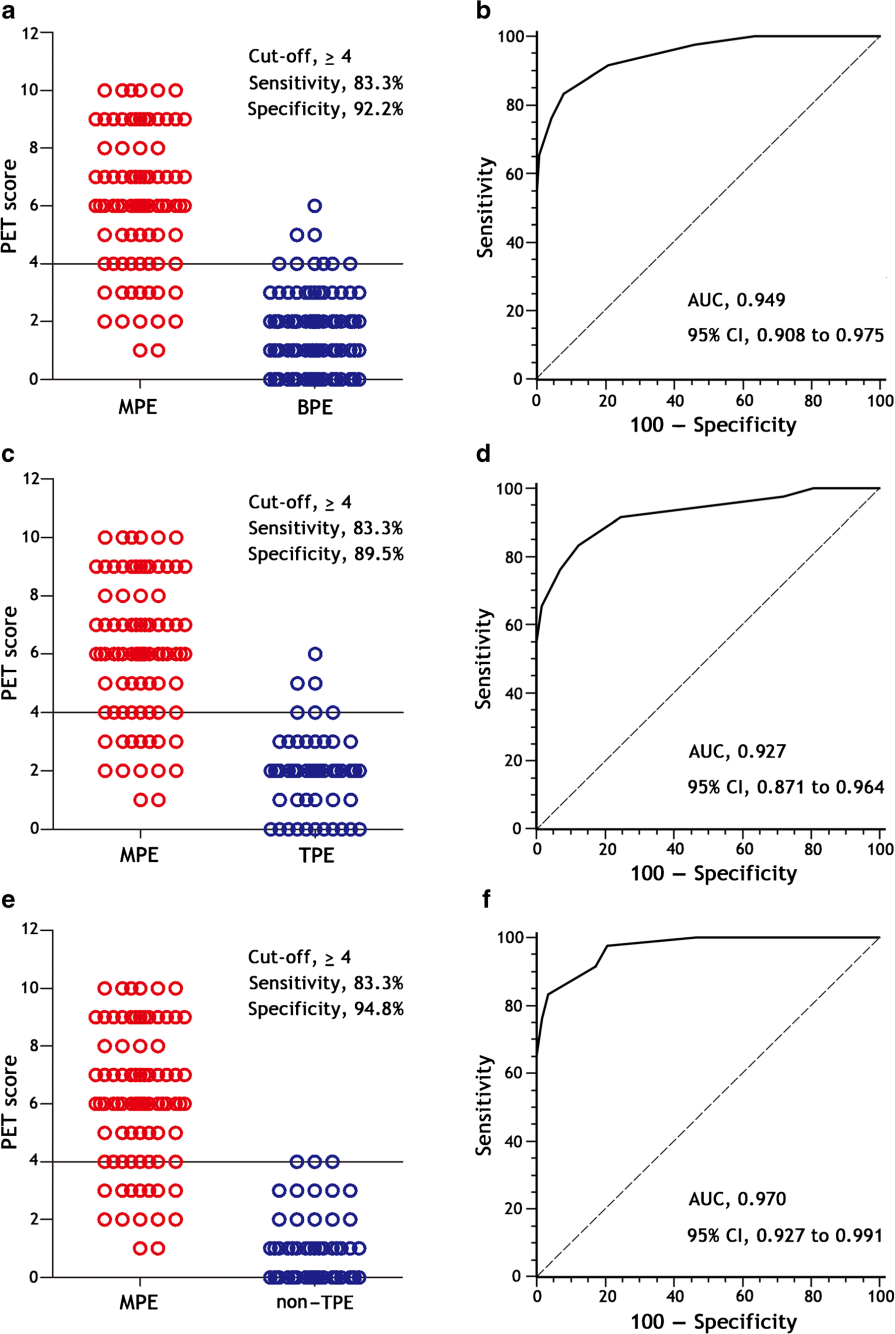
Diagnostic accuracy of the PET-CT score for the diagnosis of patients with malignant pleural effusions (MPEs) in the derivation cohort. By using a cut-off value of 4 points, the PET-CT score shows high sensitivity and specificity for the differential diagnosis of MPE (N = 84) from the overall benign pleural effusions (BPE, N = 115) (**a**), tuberculous pleural effusion (TPE, N = 57) (**c**), and non-tuberculosis pleural effusion (non-TPE, N = 58) (**e**). The receiver-operating-characteristic (AUC) curve shows the diagnostic performance of the PET-CT score for the differential diagnosis of MPE from BPE (**b**), TPE (**d**), and non-TPE (**f**)

First, we explored the diagnostic accuracy of each parameter that comprises the PET-CT score in discriminating MPE from benign effusion (Table 4). Although none of these parameters were individually satisfactory for diagnostic purposes in a clinical setting there were meaningful results when examined as a group. At the best cut-off of 4 points, the PET-CT score yielded 83.3% sensitivity (95% CI: 73.6–90.6%), 92.2% specificity (85.7–96.4%), 10.7 positive likelihood ratio (5.6–20.1), 0.2 negative likelihood ratio (0.1–0.3), and AUC 0.949 (0.908–0.975) (Table 5 and Fig. 2a and b), indicating that the PET-CT score provides acceptable differential diagnostic accuracy for patients with MPE and performs significantly better than any single PET-CT parameter.

**Table 4:** Diagnostic value of individual PET-CT parameter for malignant pleural effusion in the derivation cohort

Variable	AUC (95% CI)	Sensitivity, % (95% CI)	Specificity, % (95% CI)	PLR, (95% CI)	NLR, (95% CI)
Unilateral lung nodules and/or masses with increased ^18^F-FDG uptake (SUVmax ≥ 2.5)	0.775 (0.710–0.831)	61.9 (50.7–72.3)	93.0 (86.8–96.9)	8.9 (4.5–17.7)	0.4 (0.3–0.5)
Extrapulmonary malignancies (primary/metastatic)	0.745 (0.678–0.805)	52.4 (41.2–63.4)	96.5 (91.3–99.0)	15.1 (5.6–40.3)	0.5 (0.4–0.6)
Pleural thickening (≥3 mm) with increased ^18^F-FDG uptake (TBR > 1.8)	0.744 (0.678–0.803)	84.5 (75.0–91.5)	64.4 (54.9–73.1)	2.4 (1.8–3.1)	0.2 (0.1–0.4)
Multiple nodules or masses (uni- or bilateral lungs) with increased ^18^F-FDG uptake (SUVmax ≥ 2.5)	0.706 (0.637–0.768)	46.4 (35.5–57.6)	94.8 (89.0–98.1)	8.9 (4.0–20.0)	0.6 (0.5–0.7)
Increased pleural effusion ^18^F-FDG uptake (TBR > 1.1)	0.604 (0.532–0.672)	51.2 (40.0–62.3)	69.6 (60.3–77.8)	1.7 (1.2–2.4)	0.7 (0.5–0.9)

*AUC* area under the receiver operating characteristic curve, *CI* confidence interval, ^*18*^*F-FDG*^18^F-fluorodeoxyglucose, *NLR* negative likelihood ratio, *PLR* positive likelihood ratio, *TBR* target-to-background ratio

**Table 5:** Diagnostic value of the PET-CT score for malignant pleural effusion in the derivation cohort

The PET-CT score	Sensitivity, % (95% CI)	Specificity, % (95% CI)	PLR, (95% CI)	NLR, (95% CI)
≥3	91.7 (83.6–96.6)	80.9 (72.5–87.6)	4.8 (3.3–7.0)	0.1 (0.1–0.2)
≥4	83.3 (73.6–90.6)	92.2 (85.7–96.4)	10.7 (5.6–20.1)	0.2 (0.1–0.3)
≥5	73.8 (63.1–82.8)	97.4 (92.6–99.5)	28.3 (9.2–87.1)	0.3 (0.2–0.4)
≥6	65.5 (54.3–75.5)	99.1 (95.3–100.0)	75.3 (10.6–533.3)	0.4 (0.3–0.5)

*CI* confidence interval, *NLR* negative likelihood ratio, *PLR* positive likelihood ratio

Next, we separately analyzed the diagnostic accuracy of the PET-CT score in differentiating MPE from tuberculous effusion and from non-tuberculosis effusion. By applying the same cut-off value, we noted that the sensitivity, specificity, positive likelihood ratio, negative likelihood ratio, and the AUC of the PET-CT score in differentiating MPE (*n* = 84) from tuberculous effusion (*n* = 57) were 83.3% (73.6–90.6%), 89.5% (78.5–96.0%), 7.9 (3.7–17.0), 0.2 (0.1–0.3), and 0.927 (0.871–0.964), respectively (Fig. 2c and d), and that those for differentiating MPE from non-tuberculosis effusion (*n* = 58) were 83.3% (73.6–90.6%), 94.8% (85.6–98.9%), 16.1 (5.3–48.7), 0.2 (0.1–0.3), and 0.970 (0.927–0.991), respectively (Fig. 2e and f). We also noted that the AUC of the PET-CT score in the differential diagnosis of MPE from tuberculous effusion was similar to that of non-tuberculosis effusion, with no statistical difference (z = 1.870, *p* = 0.969), and both AUCs did not differ from that of the PET-CT score in discriminating MPE from overall benign effusion (z = 0.880 and 1.105; *p* = 0.811 and 0.864, respectively).

### Validation of the PET-CT score in the validation cohort

**Fig. 3:**
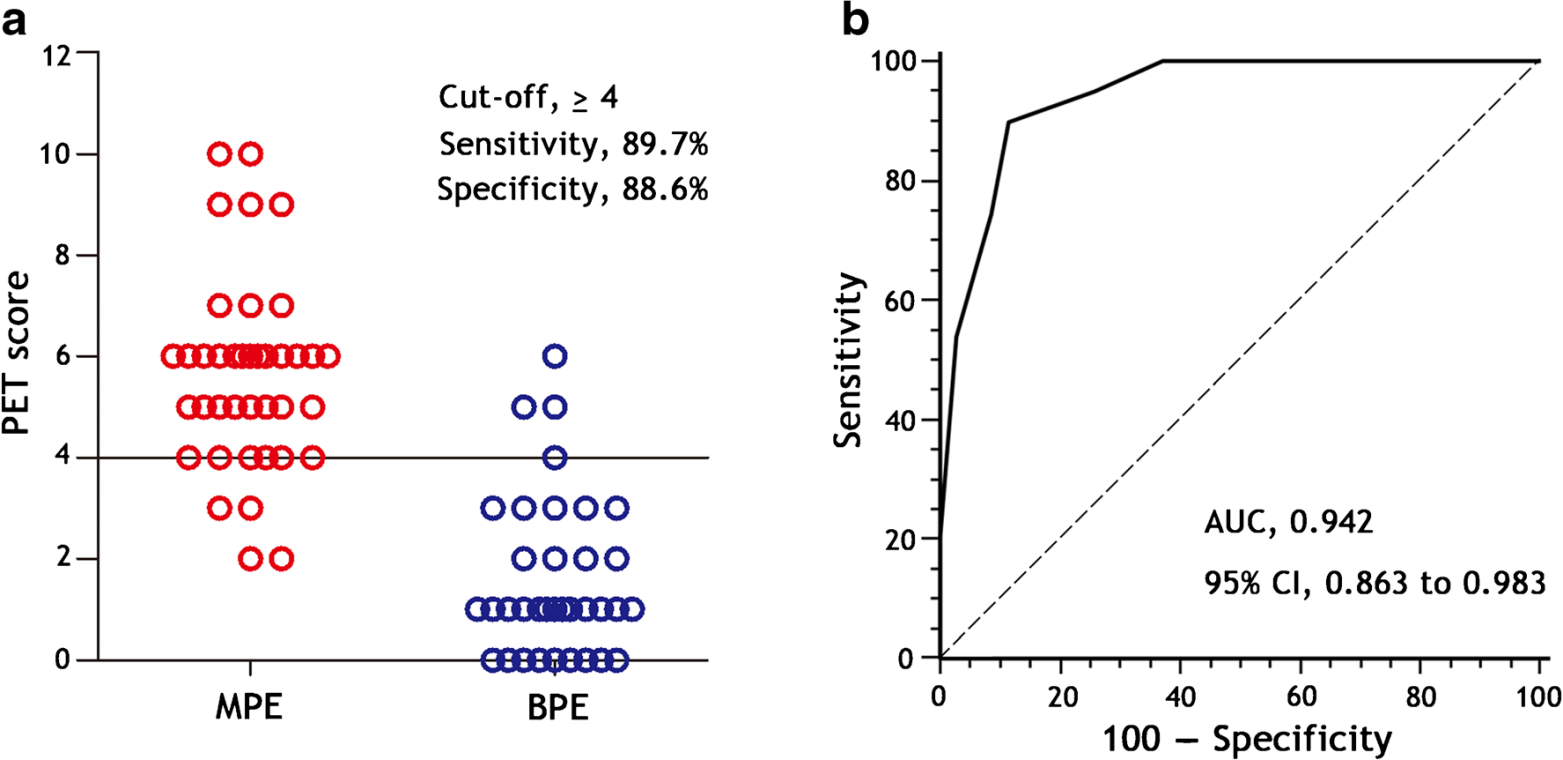
Diagnostic accuracy of the PET-CT score for the diagnosis of patients with malignant pleural effusions (MPEs) in the validation cohort. In Panel **a**, the use of a cut-off value of 4 points of the PET-CT score shows high sensitivity and specificity for the differential diagnosis of MPE (*n* = 39) from benign pleural effusions (BPE, *n* = 35). In Panel **b**, receiver operating characteristic (AUC) curve shows the diagnostic performance of the PET-CT score from the validation cohort

We performed another blinded validation study in an independent population (39 MPE and 35 benign effusions) to validate the diagnostic accuracy of a PET-CT score ≥ 4 in discriminating MPE from benign effusion. Figure 3 shows that the acceptable discrimination between MPE and benign effusion was confirmed in the validation cohort: sensitivity 89.7% (95% CI: 75.8–97.1%), specificity 88.6% (73.3–96.8%), positive likelihood ratio 7.9 (3.1–19.9), negative likelihood ratio 0.1 (0.1–0.3), and AUC 0.942 (0.863–0.983), respectively.

## Discussion

This is the largest prospective study that has investigated the diagnostic accuracy of PET-CT for patients with MPE. Based on a derivation study and a validation study, we propose a simple and feasible PET-CT scoring system with high reliability that can accurately discriminate MPE from benign effusion, as reflected by an AUC of 0.949. Our data show that from a maximum sum of 10 points of the PET-CT score, a score of ≥4 would prompt consideration of an MPE, whereas a score of <4 would mitigate against such a consideration.

Since Bury et al. first reported the application of ^18^F-FDG PET in diagnosing MPE in 1997 [7], the diagnostic accuracy of PET or PET-CT has been extensively studied; however, its exact clinical significance remains controversial. PET is based on the differential metabolism of normal and abnormal tissues, and the uptake of ^18^F-FDG is usually accelerated in tumor cells. Because some pleural inflammatory and infectious lesions can also induce increased ^18^F-FDG uptake, the underlying diseases may display false-positive findings, leading to a low diagnostic accuracy for PET-CT. To improve diagnostic ability, various methods of interpreting PET-CT, including the use of SUV threshold, ratio of SUV of pleural lesions to that of mediastinum, and dual-time-point PET have been proposed [9, 10, 17, 18]. However, the concern about false-negative results still remains.

The results from a recent meta-analysis suggest that the pooled sensitivity and specificity are 81% and 74%, respectively [12]; thus, the diagnostic performance of PET-CT for MPE is not good enough to be recommended in the routine workup of pleural effusions of undetermined causes [5]. Although our findings support previous works indicating that some PET-CT parameters are somehow helpful in the diagnosis of MPE, any single parameter does not offer sufficient evidence for its implementation in the clinical practice. Our present study provides strong evidence to support that the PET-CT score is a valuable diagnostic tool in discriminating MPE from benign effusion, with a sensitivity and specificity of 83.3% and 92.2%, respectively, from the derivation cohort. A positive likelihood ratio of 10.7 with the PET-CT score suggests that patients with MPE have about 11-fold higher chance of having a PET-CT score ≥ 4 compared to those without the disease, and this is high enough for diagnostic purposes. Moreover, such good diagnostic performance found in the derivation cohort was verified prospectively in the validation cohort. The PET-CT score system developed by this study comprehensively integrated PET and CT information, and might be better than high end CT alone [19].

The five variables that comprise the PET-CT score are readily available on PET-CT scans and have highly significant associations with the diagnosis of MPE from multivariable analysis. ^18^F-FDG PET-CT has been widely accepted for the diagnosis of lung cancer in patients with suspicious lung nodules/masses [20, 21]. Most patients with MPE in this study have lung cancer (77.4% in the derivation cohort), and these patients usually show unilateral lung nodules and/or masses with increased ^18^F-FDG uptake. In contrast, most patients with benign effusion suffered from tuberculosis and parapneumonic effusion/empyema (92.2%) without pulmonary nodules and masses. It is reasonable to infer that the presence of unilateral lung nodules and/or masses with increased ^18^F-FDG uptake is the first single variable that makes up the PET-CT score with 3 points.

Compared with other imaging examinations, one distinct advantage of PET-CT is that it may simultaneously detect malignancies occurring in any organs. If PET-CT suggests primary or metastatic tumors outside the lung, then the possibility of MPE is greatly increased. Therefore, the finding of extrapulmonary malignancies indicates the malignant nature of pleural effusion and can also be offered 3 points.

The majority (84.5%) of our patients with MPE had pleural thickening coupled with increased ^18^F-FDG uptake; whereas 73.0% of our patients with benign effusion also exhibited pleural thickening. However, only 35.7% of these patients had increased ^18^F-FDG uptake in the thickened pleura. Pleural thickening (≥3 mm) with increased ^18^F-FDG uptake enters the PET-CT score with 2 points.

Multiple pulmonary nodules or masses with increased ^18^F-FDG uptake are frequently seen in lung cancer with intrapulmonary metastasis, and sometimes can also be observed in benign diseases, such as pulmonary tuberculosis. Both malignant cells and inflammatory cells are associated with increased ^18^F-FDG uptake, and in general the former is higher than the latter [22, 23]. In the present study, multiple nodules or masses (uni- or bilateral lungs) with increased ^18^F-FDG uptake or increased pleural effusion ^18^F-FDG uptake are meaningful in differentiating MPE from benign effusion, although the coefficient is not very high (1 point each).

The most important strength of this study is its large sample size and the prospective nature of the consecutive cases in the derivation and validation cohorts. Another strength is that we conducted a comprehensive follow-up to ascertain definite etiological diagnosis of pleural effusion. However, our study had several limitations. First, in the derivation cohort, all consecutive patients with pleural effusions admitted to the Department of Respiratory and Critical Care Medicine of our hospital were recruited in the present study. All patients were persuaded to undergo PET-CT, and only those with a definite cause of pleural effusion were included in the final statistical analysis. It should be noted that some patients with a relatively easy clinical diagnosis, including 46 patients with parapneumonic effusion and three empyema, underwent PET-CT. Therefore, there might be a selection bias in the present study.

Second, we developed the PET-CT score in the high tuberculosis prevalence setting [24, 25]. It has been reported that patients with tuberculosis may show intense ^18^F-FDG uptake mimicking malignant mesothelioma [26]. A meta-analysis [27] showed that the accuracy of ^18^F-FDG PET in diagnosing lung nodules is extremely heterogeneous, and that the use of PET is less specific in diagnosing malignancy in populations with endemic infectious lung disease compared to nonendemic regions. In the present study, we separately explored the discriminative properties of the PET-CT score in identifying MPE from tuberculous and from non-tuberculosis effusions, and found the diagnostic accuracy of the PET-CT score is almost robust. Although 70.2% tuberculous patients in this study showed intense pleural uptake, 20 (35.1%) of them had only tuberculous lesions and another 31 patients (54.4%) had non-nodule pulmonary lesions (Supplemental Table 2). Therefore, according to the PET-CT score system developed by this study, their PET-CT score did not reach 4 points and then would not be considered as malignant. Therefore, the present study demonstrated the PET-CT score system can steadily identify pleural tuberculosis from malignance by a comprehensive analysis of pleura, effusion, pulmonary and extra-pulmonary lesions. Nevertheless, this finding warrants further studies.

Third, the small proportion of pleural malignant mesothelioma cases found in the present study and in our previous studies [24, 28] reflects the low incidence of this tumor in China. Tumor metastasis outside the thoracic cavity is uncommon in mesothelioma; patients with mesothelioma usually can only obtain at most 3 points (pleural thickening [≥3 mm] with increased ^18^F-FDG uptake [2 points] plus increased pleural effusion ^18^F-FDG uptake [1 point]), possibly leading to false negative results. In addition, given that our hospital is a respiratory disease-predominant general hospital, the majority of patients with MPE admitted to our institute are lung cancer patients, and that the PET-CT score is based mainly on findings outside the pleura, diagnostic accuracy of the PET-CT score in the subgroup of MPE patients caused by non-lung tumors needs further investigation.

In conclusion, we have provided evidence to support that the PET-CT score can be reliably used in the differential diagnosis of MPE. Further endorsement with prospective studies from populations with lower tuberculosis burden and/or higher incidence of non-lung malignancies, including pleural mesothelioma, would be beneficial before the PET-CT score is introduced into standard clinical practice.

## Electronic supplementary material

ESM 1 (DOCX 17.9 kb)

ESM 2 (DOCX 17.2 kb)
