# Supplementary material for: Development and validation of the PET-CT score for diagnosis of malignant pleural effusion
Source: Eur J Nucl Med Mol Imaging. 2019 Mar 22;46(7):1457–67. doi: 10.1007/s00259-019-04287-7 (PMC6533224; doi:10.1007/s00259-019-04287-7)
Supplement: Supplementary file 1 — (DOCX 17.9 kb) [file 259_2019_4287_MOESM1_ESM.docx]

**Supplemental Table 1** Characteristics of the excluded patients (N, %)

| Characteristics | Derivation group  (n=41) | Validation group  (n=39) |
| --- | --- | --- |
| Age, year | 66.3±12.4 | 63.6±16.5 |
| Sex |  |  |
| Male | 26 (63.4) | 23 (59.0) |
| Female | 15 (36.6) | 16 (41.0) |
| Excluded reasons |  |  |
| Suspected benign effusion but follow-up < 12 months | 15 (36.6) | 7 (17.9) |
| Malignant primary disease but no definite diagnosis of the plerual effusion or pleura | 18 (43.9) | 14 (35.9) |
| No confirmed primary disease and no definite diagnosis of the effusion or pleura | 8 (19.5) | 18 (46.2) |
| Referring department |  |  |
| Respiratory and Critical Care Medicine | 41 (100.0) | 29 (74.4) |
| Other departments | 0 (0.0) | 10 (25.6) |
| Diagnostic work-up |  |  |
| Serum tumor marker | 41 (100.0) | 37 (94.9) |
| Sputum | 20 (48.8) | 14 (35.8) |
| Pleural biopsy | 12 (29.2) | 6 (15.4) |
| Pleural effusion | 15 (36.6) | 9 (23.1) |
| Bronchoscopy | 13 (31.7) | 8 (20.5) |
| Biopsy of lung, lymph node or other tissues | 5 (12.2) | 7 (17.9) |
| Others (T-SPOT, PPD, etc.) | 10 (24.4) | 6 (15.4) |
