# Supplementary material for: Development and validation of the PET-CT score for diagnosis of malignant pleural effusion
Source: Eur J Nucl Med Mol Imaging. 2019 Mar 22;46(7):1457–67. doi: 10.1007/s00259-019-04287-7 (PMC6533224; doi:10.1007/s00259-019-04287-7)
Supplement: Supplementary file 2 — (DOCX 17.2 kb) [file 259_2019_4287_MOESM2_ESM.docx]

**Supplemental Table 2** Characteristics of patients with tuberculous effusion (N, %)

| Characteristics | Results |
| --- | --- |
| Time of diagnosis |  |
| Before PET-CT scan | 9 (15.8) |
| After PET-CT scan | 48 (84.2) |
| Ongoing anti-tuberculous therapy | 11 (19.3) |
| PET-CT score |  |
| (1) Pleural thickening (≥3 mm) with increased ^18^F-FDG uptake (TBR>1.8) | 40 (70.2) |
| (2) Increased pleural effusion ^18^F-FDG uptake (TBR>1.1) | 16 (28.1) |
| (3) Multiple nodules or masses (uni- or bilateral lungs) with increased ^18^F-FDG uptake (SUVmax ≥2.5) | 3 (5.3) |
| (4) Unilateral lung nodules and/or masses with increased ^18^F-FDG uptake (SUVmax ≥2.5) | 4 (7.0) |
| (5) Extrapulmonary malignancies (primary/metastatic) | 1 (1.8) |
